# Collapsed endograft and lower limb ischemia from type B dissection repaired with thoracic endovascular aortic graft and iliac stenting: A case report and review of the literature

**DOI:** 10.1016/j.jvscit.2022.02.010

**Published:** 2022-03-09

**Authors:** Peter V. Cooke, Halbert Bai, Justin M. George, Michael L. Marin, Rami O. Tadros

**Affiliations:** Division of Vascular Surgery- Department of Surgery, Icahn School of Medicine at Mount Sinai, New York, NY

**Keywords:** Thoracic aortic endovascular repair, Type B dissection, Endograft collapse

## Abstract

The collapse of an abdominal aortic endograft is a rare event. We present the case of a 60-year-old man with an abdominal endograft who came to the emergency department with chest, back, abdominal, and lower extremity pain in addition to a cool left foot. On imaging, he was found to have a type B aortic dissection and a collapsed abdominal endograft. Subsequently, the patient was taken to the operating room and treated with a thoracic endovascular aortic repair, abdominal aortic cuff, and an iliac stent. Our study details this case and thoroughly reviews similar cases in the literature.

Aortic dissection is a rare, life-threatening event with an annual incidence of approximately 6 cases per 100,000.^1^ Type B acute aortic dissections (TBAD) account for 25% to 40% of all aortic dissections.^1^ Thoracic endovascular aortic repair (TEVAR) promotes highly effective aortic remodeling in acute and subacute TBAD.^2^ Although collapsed thoracic aortic endografts have been well-documented,^3^ abdominal aortic stent graft collapse is quite infrequent. We present the rare case of a TBAD-induced collapse of an infrarenal endovascular aortic repair (EVAR), resulting in lower limb ischemia, successfully treated with TEVAR, an abdominal aortic cuff, and iliac stenting.

## Case report

Informed consent was obtained for the publication and presentation of this case report and associated images.

**Fig 1:**
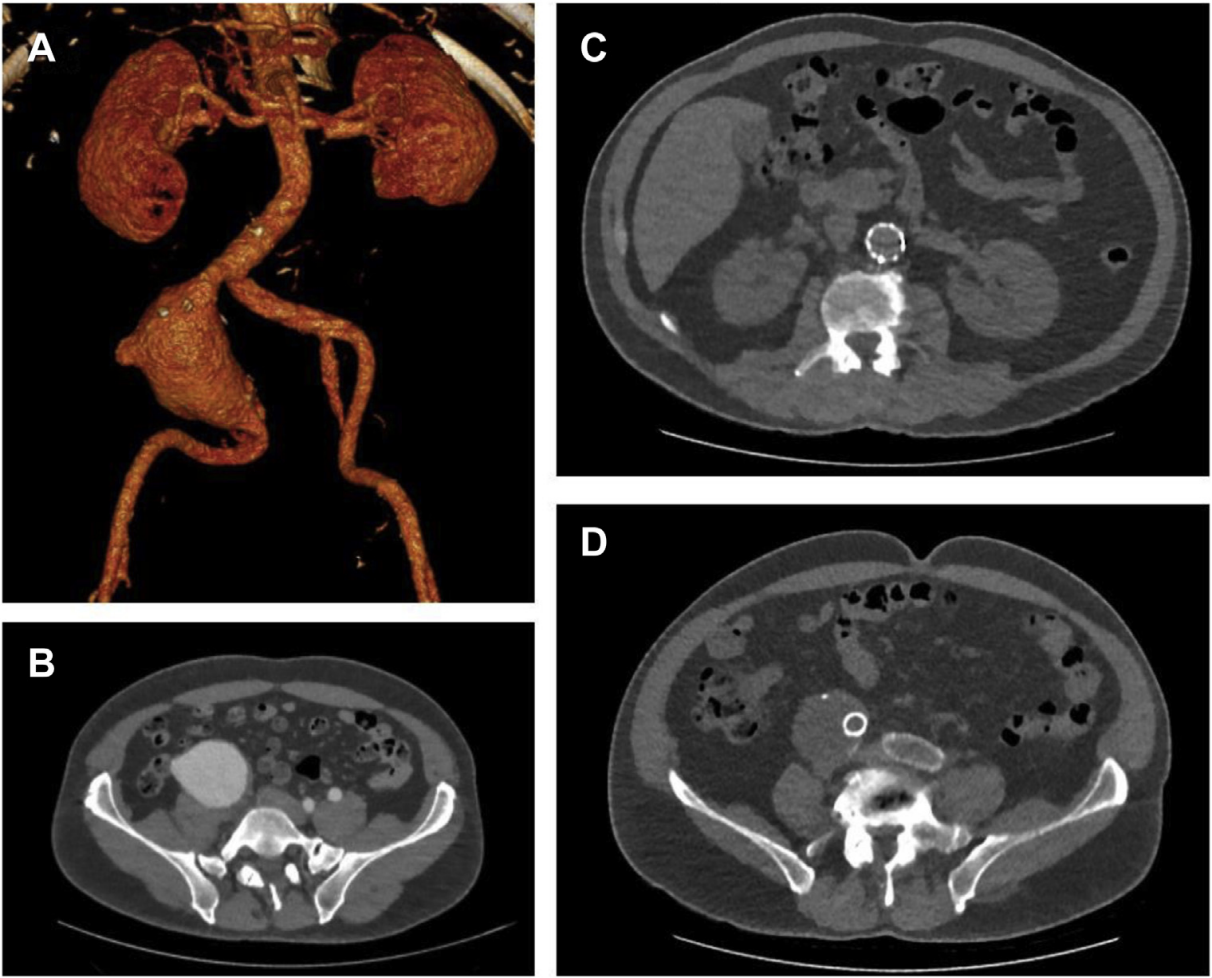
**A,** Preoperative three-dimensional reconstruction of the patient's original common iliac artery fusiform aneurysm, measuring 7.4 cm, also seen in **B,** axial view. **C,** One-year after endovascular aneurysm repair, follow-up imaging shows intact endograft in the infrarenal aorta and **D,** the common iliac artery with decrease in aneurysm sac size to 5.6 cm.

A 60-year-old man presented with a history of a right common iliac artery aneurysm with a diameter of 7.4 cm status post-EVAR with a modular aortic stent-graft device (Excluder; W. L. Gore & Assoc., Flagstaff, AZ). At his one-year follow-up, imaging revealed no endoleak or other abnormality, and his common iliac artery aneurysm had reduced to a diameter of 5.6 cm. Preoperative and one-year follow-up imaging are featured in Fig 1. Four months later (16 months after EVAR), the patient presented with ten days of chest, back, abdominal, and bilateral lower extremity pain. His blood pressure was elevated at 152/85 mm Hg and his examination was notable for a cool left foot with decreased sensation to the left ankle. Pedal Doppler signals were present and there were no signs of ischemic discoloration or skin mottling.

### Imaging

**Fig 2:**
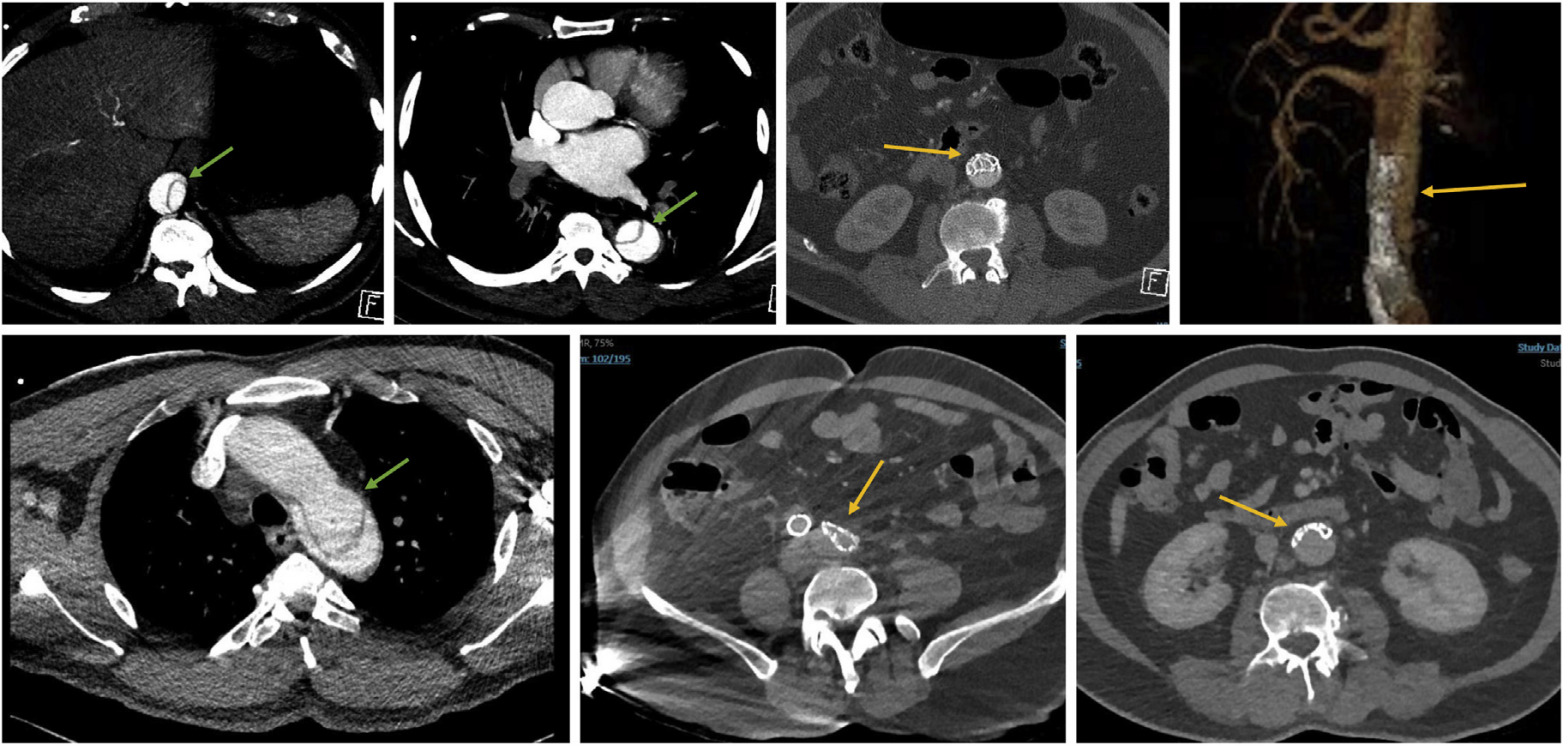
Preoperative computed tomography angiography reveals a type B acute aortic dissection (TBAD) and collapsed infrarenal endograft. Yellow arrows indicate the collapsed infrarenal endograft. *Green arrows* indicate the TBAD, beginning at the level of the left common carotid artery.

A computed tomography angiogram revealed a thoracic aortic dissection originating from just distal to the left common carotid artery extending to the left common femoral artery. The infrarenal aortic graft and the left iliac distal landing zone were compressed by the false lumen of the dissection (Fig 2). The proximal descending aorta was dilated to 4.5 cm. There were several high-risk elements of the dissection, including an entry tear in the inner aortic curvature of more than 10 mm, a descending false lumen diameter or more than 22 mm, and a patent, round false lumen with an elliptical true lumen.

The patient was started on esmolol drip for blood pressure control. A TEVAR with prophylactic left subclavian revascularization was planned.

### Operation

The patient underwent left carotid artery to left subclavian artery bypass in preparation for concomitant zone 2 TEVAR deployment.

Bilateral femoral access was obtained under ultrasound guidance. Using the preclose technique, two ProGlide devices were deployed on the right. From the right femoral access, using a glidewire and glide catheter combination, the collapsed stent graft was traversed successfully. The wire position in the true lumen was confirmed with intravascular ultrasound examination and angiography. A Lunderquist wire was then positioned in the ascending aorta. From the left iliac limb, a glide wire could not be passed through the stent graft because it was crushed from the pressurized false lumen. From the right access, a compliant balloon was used to open the abdominal graft (Fig 2), allowing the glide wire to pass on the left side. Of note, if the infrarenal aortic stent graft could not be traversed from the femoral access, we were prepared for an antegrade approach from the right arm given the patient's left subclavian artery embolization. After crossing antegrade, we would snare the wire, allowing delivery of the large-bore device through the femoral access.

Access through the graft bilaterally was obtained. A 38 × 217 mm Cook Zenith Alpha Thoracic Endovascular Graft (Cook ZTA) (Cook Medical, Bloomington, IN) was deployed in zone 2 to address the entry tear. Given continued true lumen compression, an extension was placed using a 38 × 167 mm Cook ZTA, landing just proximal to the origin of the celiac artery. A postdeployment angiogram revealed improved true lumen diameter with some regression of the false lumen and no evidence of retrograde type A dissection. A Cook 32 × 39 mm aortic cuff was deployed just below the renal arteries to provide additional radial force to the infrarenal device and prevent recollapse in the setting of false lumen thrombosis and remodeling.

An aortoiliac angiogram demonstrated continued fixed obstruction of the left iliac artery. A Gore Viabahn Balloon Expandable 11 × 79 mm stent graft was used to reline the left iliac limb. A completion angiogram demonstrated coverage of the dissection entry tear and patency of the infrarenal device with filling of the bilateral iliac limbs. Postoperatively, the patient had palpable pedal pulses and resolution of leg pain.

### Postoperative course

**Fig 3:**
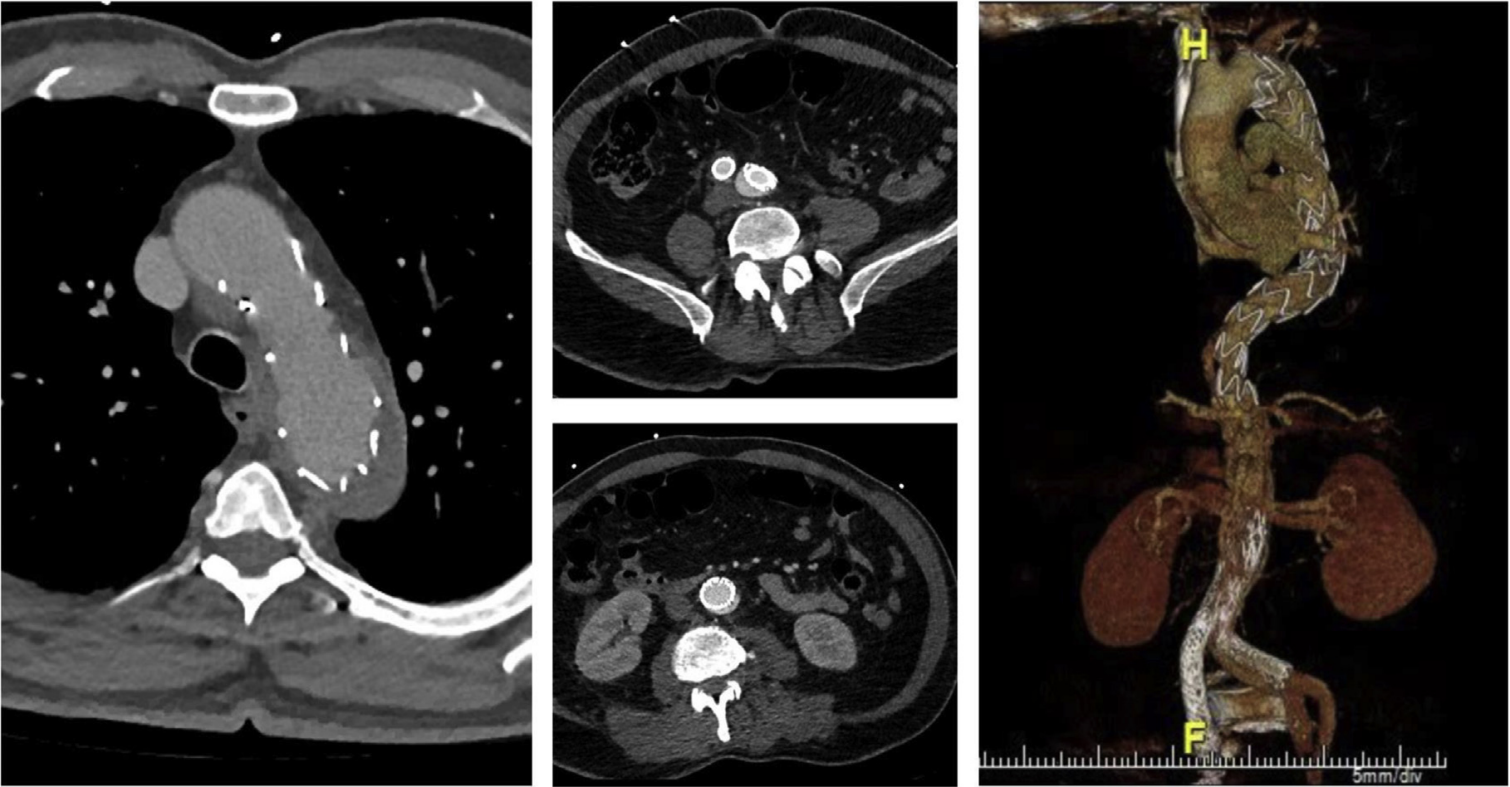
Postoperative computed tomography angiography shows remodeling of the thoracic aorta and reexpansion of the infrarenal endograft with reinforcement from an aortic cuff. Sufficient perfusion to the visceral and iliac arteries is visualized.

The patient was discharged on his normal home regimen of aspirin, atorvastatin, and blood pressure medications. A computed tomography angiogram at the 2-week postoperative assessment demonstrated favorable remodeling with false lumen thrombosis (Fig 3). The patient was able to resume his daily activities.

## Discussion

Stent graft collapse is a rare complication after EVAR. Other etiologies beyond TBAD for stent graft collapse have included angulation of the aortic neck, oversized endografts, stent migration, and type A dissection.^4^ Similar cases of collapsed abdominal EVAR stent grafts from TBAD have been sparsely reported in the literature. Of the 12 reported cases we found, 11 patients (92%) were male and the mean patient age was 70.8 years. The median time to presentation after the abdominal EVAR was 13 months. Patients presented most frequently with acute abdominal pain, limb ischemia, back pain, and/or paraplegia. Four patients (33%) died en route to the hospital or shortly after reoperation. These 12 cases are summarized in the Table.^5^, ^6^, ^7^, ^8^, ^9^, ^10^, ^11^, ^12^, ^13^, ^14^, ^15^

**Table:** Characteristics of type B acute aortic dissection-induced endovascular aneurysm repair collapse in the literature

Year published/first author	Age/sex	Collapsed device	Original graft location	Reason for EVAR collapse	Time with first EVAR before collapse	Repair strategy	Symptoms	Outcome
2003/Haulon et al^5^	53/F	Excluder (Gore)	AAA	TBAD	6 months	Patient died en route	Bilateral lower extremity pain, parathesia, cardiac arrest	Dead
2009/Iyer et al^6^	77/M	Zenith Flex (Cook)	Infrarenal AAA	TBAD	11 weeks	TEVAR and thrombectomy	Upper abdominal and back pain, bilateral lower extremity ischemia	Alive
2009/van Keulen et al^7^	74/M	Talent (Medtronic)	AAA	TBAD	1.5 years	TEVAR and stent placement for collapsed endograft main body	Abdominal pain	Alive
2014/Psacharopulo et al^8^	75/M	Excluder (Gore)	AAA	TBAD	14 months	Axillofemoral bypass	Abdominal and back pain, bilateral lower extremity ischemia	Dead
2014/Vainas et al^9^	88/M	Zenith (Cook)	Infrarenal AAA	TBAD	10 years	Axillofemoral bypass →open abdominal aortic repair and TEVAR	Bilateral lower extremity pain	Alive
2015/Yoshiga et al^10^	69/M	Endurant II	AAA	TBAD	6 months	TEVAR and iliac leg insertion	Thoracoabdominal pain	Alive
2017/Goto et al^11^	65/M	Excluder (Gore)	AAA	TBAD	2 years	Axillofemoral bypass	Paraplegia	Alive
2017/Itoga et al^12^	59/M	Excluder (Gore)	Infrarenal AAA	TBAD	4 years	TEVAR and thrombectomy	Bilateral buttock stabbing discomfort	Alive
2018/Nomura et al^13^	73/M (patient 1) 83/M (patient 2)	Zenith (Cook) (patient 1) Excluder (Gore) (patient 2)	AAA (patients 1 and 2)	TBAD (patients 1 and 2)	7 years (patient 1) 6 months (patient 2)	Died in ambulance (patient 1) extra-anatomical bypass (patient 2)	Lower back pain (patient 1), back pain and lower limb ischemia (patient 2)	Dead (patients 1 and 2)
2019/Ostapenko et al^14^	71/M	Zenith (Cook)	AAA	TBAD	Several years	TEVAR	Chest and back pain with left lower extremity ischemia	Alive
2020/Motoji et al^15^	62/M	Excluder (Gore)	Infrarenal AAA	TBAD	3 years	Axillofemoral bypass →open abdominal aortic repair	Acute back pain and paresthesia of the lower limbs	Alive

*AAA,* Abdominal aortic aneurysm; *TEVAR,* thoracic endovascular aortic repair.

The etiology of TBAD in patients with abdominal EVAR remains unknown. We hypothesize that our case was a primary TBAD, with a high-pressured false lumen compressing and collapsing the abdominal aortic stent graft. The abdominal endograft may have prevented a reentry tear, leading to a mean false lumen pressure that was much greater than the true lumen pressure.^4^^,^^8^ Furthermore, the remodeling of an undetected aortic dissection may be a precursor for infrarenal AAA and the two events may not always be mutually exclusive.^16^ Although the stent graft material might play a role in susceptibility of collapse, the number of reported cases is too small to draw definitive conclusions. Both nitinol, as in the current case, and stainless steel^6^ devices have been reported to collapse. TEVAR collapse has also been reported with a median time to graft collapse within the first 2 postoperative weeks.^3^ High radial force devices have been used to repair these collapsed thoracic grafts. Further research is necessary to determine the precise etiology and incidence of TBAD-induced abdominal stent graft collapse.

A consensus on treatment strategies for abdominal EVAR collapse has not been established. Previously reported successful repair strategies include axillofemoral bypass,^9^^,^^11^^,^^15^ open abdominal aortic repair,^9^^,^^15^ and TEVAR of the proximal tear.^6^^,^^7^^,^^10^^,^^12^^,^^15^ The endovascular treatment of abdominal endograft collapse, generally with an aortic balloon and/or cuff, is a viable approach.^6^^,^^7^ In our case, an aortic cuff was placed just below the level of the renal arteries. We did not believe that balloon remodeling alone would suffice for long-term fixation of the stent graft in the setting of continued aortic remodeling.

After TEVAR and infrarenal aortic cuff placement on the Gore Excluder device, perfusion in our patient's left common iliac artery was still compromised; thus, we elected to place a Viabahn Balloon Expandable 11 × 79 mm graft. Iliac stenting has been reported for the treatment of limb ischemia in other cases of aortic stent graft collapse.^10^^,^^14^ Balloon thromboembolectomy has also been described to reline the iliac limb of the collapsed EVAR.^12^ In contrast, in some reports of EVAR collapse with severe lower extremity ischemia, normal perfusion of the iliac arteries has returned simply after reestablishing the structure of the aortic stent graft.^6^ This treatment discrepancy suggests that the use of iliac stents should be an intraoperative case-dependent decision in these clinical scenarios.

## Conclusions

TBAD in patients with a previous EVAR can lead to a collapse of the endograft and subsequent lower extremity ischemia. Our case describes the management of this pathology with ballooning to reopen the EVAR, TEVAR to repair the entry tear, an aortic cuff to reinforce the infrarenal graft, and iliac stenting to restore proper lower extremity perfusion. With increasing EVAR use and life expectancy, the incidence of this unique problem is likely to increase.

## References

[1] Howard D.P., Banerjee A., Fairhead J.F., Perkins J., Silver L.E., Rothwell P.M. (2013). Population-based study of incidence and outcome of acute aortic dissection and premorbid risk factor control: 10-year results from the Oxford Vascular Study. Circulation.

[2] Tadros R.O., Tang G.H.L., Barnes H.J., Mousavi I., Kovacic J.C., Faries P. (2019). Optimal treatment of uncomplicated type B aortic dissection: JACC review topic of the week. J Am Coll Cardiol.

[3] Tadros R.O., Lipsitz E.C., Chaer R.A., Faries P.L., Marin M.L., Cho J.S. (2011). A multicenter experience of the management of collapsed thoracic endografts. J Vasc Surg.

[4] Rathore A., Gloviczki P., Oderich G.S., Bower T.C. (2019). Collapsed bifurcated modular infrarenal endograft. J Vasc Surg.

[5] Haulon S., Greenberg R.K., Khwaja J., Turc A., Srivastava S.D., Eagleton M. (2003). Aortic dissection in the setting of an infrarenal endoprosthesis: a fatal combination. J Vasc Surg.

[6] Iyer V., Rigby M., Vrabec G., Sr (2009). Type B aortic dissection after endovascular abdominal aortic aneurysm repair causing endograft collapse and severe malperfusion. J Vasc Surg.

[7] van Keulen J.W., Toorop R.J., de Borst G.J., Scharn D.M., Prokop M., Moll F.L. (2009). Abdominal stent-graft collapse due to progression of a Stanford type B dissection. J Endovasc Ther.

[8] Psacharopulo D., Ferrero E., Viazzo A., Ferri M., Ripepi M., Nessi F. (2014). Abdominal aortic endograft collapse due to false lumen radial force of an acute type B aortic dissection. Ann Vasc Surg.

[9] Vainas T., Tielliu I.F., Wallis de Vries B.M., van der Laan M., Zeebregts C.J., van den Dungen J.J. (2014). Acute type B dissection complicated by infrarenal aortic stent-graft collapse with spontaneous re-expansion. J Endovasc Ther.

[10] Yoshiga R., Morisaki K., Matsubara Y., Yoshiya K., Inoue K., Matsuda D. (2015). Emergency thoracic aortic stent grafting for acute complicated type B aortic dissection after a previous abdominal endovascular aneurysm repair. Surg Case Rep.

[11] Goto Y., Hosoba S., Ogawa S., Kinoshita Y. (2017). Collapsed stent graft and severe malperfusion 2 years after endovascular aortic repair. Eur J Cardiothorac Surg.

[12] Itoga N.K., Wu T., Dake M.D., Dalman R.L., Lee J.T. (2018). Acute type B dissection causing collapse of EVAR endograft and iliac limb occlusion. Ann Vasc Surg.

[13] Nomura Y., Nagao K., Hasegawa S., Kawashima M., Tsujimoto T., Izumi S. (2019). Fatal complications of new-onset complicated type B aortic dissection after endovascular abdominal aortic aneurysm repair: report of 2 cases and literature review. Vasc Endovascular Surg.

[14] Ostapenko A., Richard M.N., Dietzek A.M. (2019). Rare case of acute type B dissection causing complete collapse of EVAR stent. Vasc Endovascular Surg.

[15] Motoji Y., Kato T., Seki J., Tsumura K., Tomita S., Okawa Y. (2020). A case of collapsed stent graft, severe lower limb ischemia, and ruptured abdominal aortic aneurysm due to type B acute aortic dissection 3 years after endovascular aneurysm repair. Ann Vasc Dis.

[16] Miner G.H., Taubenfeld E., Tadros R.O., Han D.K., Marin M.L. (2020). Decreased abdominal aortic aneurysm size following EVAR in patients with CT evidence of subclinical thoracic aortic dissection. Ann Vasc Surg.

